# Investigating causal effects of income on health using two-sample Mendelian randomisation

**DOI:** 10.1186/s44263-025-00130-4

**Published:** 2025-02-10

**Authors:** Erik Igelström, Marcus R. Munafò, Ben M. Brumpton, Neil M. Davies, George Davey Smith, Pekka Martikainen, Desmond Campbell, Peter Craig, Jim Lewsey, S. Vittal Katikireddi

**Affiliations:** 1 MRC/CSO Social and Public Health Sciences Unit, School of Health and Wellbeing, University of Glasgow, Glasgow, UK; 2 MRC Integrative Epidemiology Unit (IEU), Bristol Medical School, University of Bristol, Bristol, UK; 3 Bristol Medical School, Population Health Sciences, University of Bristol, Bristol, UK; 4 K. G. Jebsen Center for Genetic Epidemiology, Department of Public Health and Nursing, Norwegian University of Science and Technology, Trondheim, Norway; 5 Department of Statistical Sciences, University College London, London, UK; 6 Population Research Unit, Faculty of Social Sciences, University of Helsinki, Helsinki, Finland; 7 The Max Planck Institute for Demographic Research, Rostock, Germany; 8 Max Planck, University of Helsinki Center for Social Inequalities in Population Health, Helsinki, Finland; 9 Health Economics and Health Technology Assessment, School of Health and Wellbeing, University of Glasgow, Glasgow, UK

**Keywords:** Mendelian randomization, Income, Health inequalities, Social determinants of health, Genetic epidemiology, Causal inference

## Abstract

**Background:**

Income is associated with many health outcomes, but it is unclear how far this reflects a causal relationship. Mendelian randomisation (MR) uses genetic variation between individuals to investigate causal effects and may overcome some of the confounding issues inherent in many observational study designs.

**Methods:**

We used two-sample MR using data from unrelated individuals to estimate the effect of log occupational income on indicators of mental health, physical health, and health-related behaviours. We investigated pleiotropy (direct effects of genotype on the outcome) using robust MR estimators, CAUSE, and multivariable MR including education as a co-exposure. We also investigated demographic factors and dynastic effects using within-family analyses, and misspecification of the primary phenotype using bidirectional MR and Steiger filtering.

**Results:**

We found that a 10% increase in income lowered the odds of depression (OR 0.92 [95% CI 0.86–0.98]), death (0.91 [0.86–0.96]), and ever-smoking (OR 0.91 [0.86–0.96]), and reduced BMI (− 0.06 SD [− 0.11, − 0.003]). We found little evidence of an effect on alcohol consumption (− 0.02 SD [− 0.01, 0.05]) or subjective wellbeing (0.02 SD [− 0.003, 0.04]), or on two negative control outcomes, childhood asthma (OR 0.99 [0.87, 1.13]) and birth weight (− 0.02 SD, [− 0.01, 0.05]). Within-family analysis and multivariable MR including education and income were imprecise, and there was substantial overlap between the genotypes associated with income and education: out of 36 genetic variants significantly associated with income, 29 were also significantly associated with education.

**Conclusions:**

MR evidence provides some limited support for causal effects of income on some mental health outcomes and health behaviours, but the lack of reliable evidence from approaches accounting for family-level confounding and potential pleiotropic effects of education places considerable caveats on this conclusion. MR may nevertheless be a useful complement to other observational study designs since its assumptions and limitations are radically different. Further research is needed using larger family-based genetic cohorts, and investigating the overlap between income and other socioeconomic measures.

**Supplementary Information:**

The online version contains supplementary material available at 10.1186/s44263-025-00130-4.

## Background

People with lower incomes tend to have poorer health outcomes, but we do not know how far this reflects a causal relationship between income and health. Previous studies have assessed whether these relationships are causal using a variety of designs, including lottery studies [1–3], trials of cash transfer programmes [4–6], longitudinal studies of within-person income changes [7–9], and evaluations of policy reforms [10–12]. These studies suggest that the causal effects of income changes might be substantially smaller than the cross-sectional income-health relationship; however, the most robust studies typically rely on relatively short-term, one-off, or otherwise idiosyncratic exposures that may not reflect the effects of changes to lifetime income. While this may be adequate for predicting the immediate effect of interventions on income, modelling the longer-term and intergenerational effects of policies that affect income requires evidence on the effects of lifetime income. These are, however, much more challenging to estimate using observational data without substantial risk of confounding and measurement error. Here we add to the literature by using a novel source of evidence about the effects of income—genetics and Mendelian randomisation (MR).

**Fig. 1:**
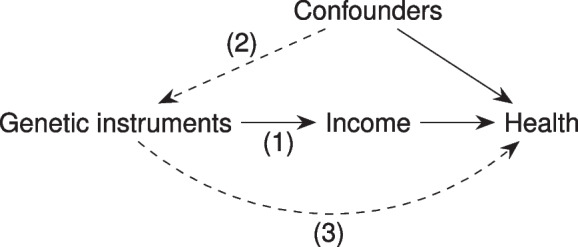
Illustration of the three instrumental variable assumptions: (**1**) relevance, (**2**) independence, and (**3**) exclusion

MR is an approach to investigating causal effects by exploiting genetic variation, generally by using genetic variants (single nucleotide polymorphisms, or SNPs) as instrumental variables for an exposure of interest. Instrumental variables are defined by three assumptions: (1) relevance, that the instrument is associated with the exposure; (2) independence, that there are no uncontrolled confounders of the instrument-outcome association; and (3) exclusion, that the instrument only affects the outcome via its effect on the exposure [13–16] (Fig. 1). MR has traditionally been used to study exposures thought to be directly related to genetics (for instance disease status), but has also been used to study more ‘biologically distal’ exposures such as education [17–20]. A genome-wide association study (GWAS) by Hill and colleagues [21] identified 68 independent genetic variants associated at genome-wide significance with household income level in the UK Biobank cohort; subsequently, Kweon and colleagues [22] identified 45 independent variants associated with individual log hourly wages. These data have recently begun to be used to represent household income as an exposure or mediator in multivariable MR (MVMR) studies [23–26], but have not yet focused on income as the primary exposure.

As a tool for investigating causal effects, MR has some potential benefits that could be important when investigating social determinants of health. First, since germline genetic variation is fixed at conception, it cannot be affected by events that occur during an individual’s lifetime. Such reverse causation is a major challenge in most other non-experimental approaches, where it is rarely possible to fully control for time-varying confounders such as changes in employment status, physical and mental health, and household structure. Second, MR estimates the effect of differences in underlying liability to an exposure, which result in different long-term exposure trajectories. Hence, the causal contrast in MR is not that of a change in exposure at the time it is measured, but a variation in genotype at conception, which would result in a different exposure trajectory throughout the lifetime [27]. The long-term, cumulative effect of income is notoriously challenging to investigate and measure using conventional epidemiological designs, as phenotypic income variables are necessarily measured at specific timepoints, but subject to large variations over time, making them a poor proxy for lifetime income [28]. MR has the potential to reduce bias due to such measurement error [29].

However, if the instrumental variable assumptions are violated, the MR estimates may be biased [20, 30, 31]. Since socioeconomic inequalities in health often persist across generations and within families, genetic instruments used in MR may plausibly be associated with health outcomes due to intergenerational confounding pathways, such as assortative mating or dynastic effects [32]; this would violate the second assumption (independence). Additionally, a genetic variant associated with income may be mediated by some heritable trait that also has direct effects on health not involving income (‘horizontal pleiotropy’); this would violate the third assumption (exclusion). In both cases, we may expect MR estimates to overestimate the true effect.

These biases are possible in any MR analysis, but could plausibly be of particular concern in the case of income. Income is strongly geographically patterned, correlated across generations, and likely to be implicated in assortative mating [33], increasing the likelihood of bias due to population stratification and dynastic effects. Additionally, many of the traits that could be expected to mediate the effect of genetic instruments on income (such as physical disabilities, cognitive ability, or personality traits) could plausibly also affect health in the same direction, meaning that horizontal pleiotropy could be expected to bias effect estimates away from the null.

Given the limitations and challenges in the existing literature on income and health, incorporating evidence from diverse and contrasting methodological approaches may help broaden our understanding of the causal mechanisms linking income and health [17, 34, 35]. MR can potentially add compelling new evidence about the effects of income on health, but it is crucial to judiciously interpret the results. The tools and data needed to conduct MR analyses are now freely and openly available, and this has prompted concerns about the risk of poor-quality studies making strong but misleading causal claims based on such analyses [36, 37].

In this paper, we propose and apply a framework for using MR to investigate the effects of income on health. We first apply conventional two-sample MR methods to income and a range of health outcomes (covering mental health, physical health, and health behaviours) to obtain our primary effect estimates. Two-sample MR uses summary data on genotype-exposure and genotype-outcome associations obtained in separate, ideally exchangeable, populations, negating the need for individual-level data [38]. We then systematically investigate several alternative pathways which could provide a non-causal explanation for the effects seen in the primary analyses: pleiotropy (using robust MR methods and MVMR including education as a co-exposure), demographic factors and dynastic effects (using within-family analyses), and misspecification of the primary phenotype (using bidirectional MR and Steiger filtering) [38]. We consider education as a co-exposure because it may be an important mediator on the pathway from SNPs to income [22], and hence a key source of pleiotropy.

We add to prior literature in two ways. First, we present novel estimates of the effect of income on health, which may provide more robust causal evidence as well as overcome many limitations of prior evidence: in particular by estimating the effect of lifetime income rather than short-term income variations. We facilitate comparison with existing literature by reporting results in terms of a 10% increase in income. Second, by systematically investigating and discussing potential sources of bias, we provide an exemplar for the application of MR to socioeconomic exposures.

## Methods

### Study design and data sources

We conducted two-sample MR analyses using publicly available summary data from separate GWASs on income, education, and ten pre-specified health outcomes across the domains of mental health, physical health, and health behaviours (Table 1). Our primary exposure was standardised individual log hourly wages, obtained from a GWAS of individual occupational wages in the UK Biobank by Kweon and colleagues [22, 39], which identified 45 independent SNPs, each explaining between 0.011% and 0.037% of the variance in income. In this GWAS, log hourly wages for UK Biobank participants were imputed using data on working hours and occupational codes, with an imputation model estimated using the UK Labour Force Survey and Annual Survey of Hours and Earnings [22]. We also included years of education [40] as a co-exposure in MVMR analyses.

**Table 1:** Genome-wide association studies used in the two-sample Mendelian randomisation analyses

Phenotype	Reference	Sample size	UK biobank overlap^a^	Unit of measurement
Income (log hourly wages)	[22]	282,963	282,963 (100%)	SD
Educational attainment (years)	[40]	766,345	442,183 (58%)	SD
**Mental health**				
Depression	[41]	218,792	0	Log odds
Anxiety disorders	[41]	218,792	0	Log odds
Subjective wellbeing	[42]	298,420	40,543 (14%)	SD
**Physical health**				
Death (all causes)	[41]	218,792	0	Log odds
Body mass index	[43]	322,154	0	SD
**Health behaviours**				
Ever smoked	[44]	249,171	0	Log odds
Cigarettes per day	[44]	143,210	0	SD
Alcohol consumption (units per week)	[44]	226,223	0	SD
**Negative control outcomes**				
Childhood asthma (onset age < 16)	[41]	218,792	0	Log odds
Birth weight	[45]	143,677	67,786 (47%)	SD

*SD* standard deviation

^a^Participant overlap between the exposure and outcome data sets can cause bias in Mendelian randomisation analyses

The outcomes consisted of three mental health measures (diagnosed depression [41], diagnosed anxiety disorder [41], and subjective wellbeing [42]), two physical health measures (all-cause mortality [41] and body mass index (BMI) [43]), and three health behaviour measures (ever smoking, alcohol consumption, and smoking intensity [44]). We also included two negative control outcomes, childhood asthma (onset before age 16) [41] and birth weight [45]; we expect these not to be directly affected by an individual’s own income, as they are measured before working age, and the purpose of including such negative control outcomes is to detect residual confounding or dynastic effects [46, 47].

For these outcomes, we searched the NHGRI-EBI GWAS Catalog [48] and OpenGWAS database [49] as described in the protocol [50] to identify the largest available GWAS for each outcome. In order to minimise bias due to sample overlap [51], we excluded studies with more than 50% sample overlap with the UK Biobank cohort, which was used for the SNP–﻿exposure associations.

We estimated results using the conventional inverse-variance weighted (IVW) MR estimator [52], and investigated potential biases using pleiotropy-robust estimators: weighted median estimator (WME) [53], weighted mode-based estimator (WMBE) [54], MR-Egger [55], and Causal Analysis Using Summary Effect estimates (CAUSE) [56]. We also conducted MVMR including education as a co-exposure, bidirectional MR, and within-family MR.

Prior to conducting analyses, a detailed study protocol was produced and registered in OSF [50]. The reporting of this study follows the ‘Strengthening The Reporting of Observational Studies in Epidemiology using Mendelian Randomisation’ (STROBE-MR) guidelines [15, 16] as shown in Additional file 1. The analysis code was shared on DeSci and GitHub [57, 58].

### Negative control outcomes

We used negative control outcomes to detect the influence of unmeasured pleiotropy and confounding (e.g. due to population stratification or dynastic/family effects). A negative control outcome should be vulnerable to similar sources of bias (e.g. confounding) as other outcomes, but also plausibly unaffected by the exposure of interest (individual occupational income); under these assumptions, any apparent effect of income on a negative control outcome can be attributed to bias [46, 47]. Since individuals generally start to earn occupational income in adulthood, childhood health outcomes are plausible negative controls. We used birth weight and childhood-onset (age < 16) asthma, which are associated with measures of parental socioeconomic position (e.g., income and education [59, 60]) that are also plausible confounders of income and health.

### Primary Mendelian randomisation analyses

We estimated the effect of income on each outcome using four MR estimators making different assumptions about possible pleiotropy. The IVW estimator assumes that there is no directional pleiotropy, that is, that any pleiotropic effects sum to zero across all SNPs used as instruments [55]. WME [53] and WMBE [54] are less biased by directional pleiotropy, under the assumption that some subset of SNPs is unaffected by pleiotropy (at least 50% of weighted SNPs for WME, and the largest set of SNPs estimating similar effects for WMBE). MR-Egger [55] allows directional pleiotropy among all SNPs, as long as the extent of pleiotropy is uncorrelated with the instruments’ strength. This is known as the Instrument Strength Independent of Direct Effect (InSIDE) assumption.

We performed two-sample MR using the *MendelianRandomization* package [61]. For each outcome, we identified all SNPs that were statistically significant in the exposure dataset (at $p<5\times {10}^{-8}$) and also present in the outcome dataset. We clumped the resulting set of SNPs using PLINK 1.90 [62], the 1000 Genomes reference population [63], an ${R}^{2}$ threshold of 0.001, and a window of 10,000 kb. To judge instrument strength, we calculated the *F*-statistic as ${\left({\widehat{\beta }}_{X}/{\text{se}}\left({\widehat{\beta }}_{X}\right)\right)}^{2}$ for each instrument-exposure association ${\widehat{\beta }}_{X}$.

The IVW, WME, WMBE, and MR-Egger estimators all rely on the instrumental variable assumptions:

1. Instrument SNPs have a causal effect on the exposure (‘relevance’)
2. Instrument SNPs affect the outcome only via the exposure (‘exclusion restriction’ or ‘no pleiotropy’)
3. There is no confounding between the instrument SNPs and the outcome

The estimators differ in how the assumptions apply across the set of instruments used. IVW assumes that the assumptions are met for all instruments, WME that they are met for a majority of instruments, and WMBE that they are met for a plurality of instruments estimating the same effect. MR-Egger allows directional pleiotropy to be present but assumes that it is uncorrelated with any exposure-outcome confounders.

We also used the CAUSE method as a further test of causality [56]. This method compares a ‘causal’ model assuming a non-zero causal effect in addition to potential pleiotropy, with a ‘sharing’ model assuming only pleiotropy and no causal effect. If the causal model fits better than the sharing model, this provides evidence of a causal effect.

More precisely, CAUSE assumes that the effect of each SNP on the outcome is a mixture of causal (mediated only by the exposure), correlated pleiotropic (mediated by some confounder of the exposure and outcome), and uncorrelated pleiotropic effects. Two nested models are estimated using Bayesian methods, one assuming that there is a non-zero causal effect (the ‘causal model’) and the other assuming zero causal effect (the ‘sharing model’). The two models are compared and a *z*-score computed, from which a p-value can be derived, with a low p-value suggesting that the causal model is a better fit. We estimated nuisance parameters using a random sample of 1,000,000 SNPs (taken from all SNPs common between the exposure and outcome GWAS) and used a significance threshold of $p<{10}^{-3}$ for the analysis.

The income GWAS originally reported coefficients in terms of a standard deviation (SD) change in log income. In order to obtain results on a more interpretable scale, we rescaled our effect estimates to represent the effect of a 10% increase, by first dividing them by the SD of log income in the GWAS sample (0.351), then multiplying by log(1.1). For binary outcomes, we then exponentiated these rescaled coefficients to obtain odds ratios.

### Multivariable Mendelian randomisation including education

**Fig. 2:**
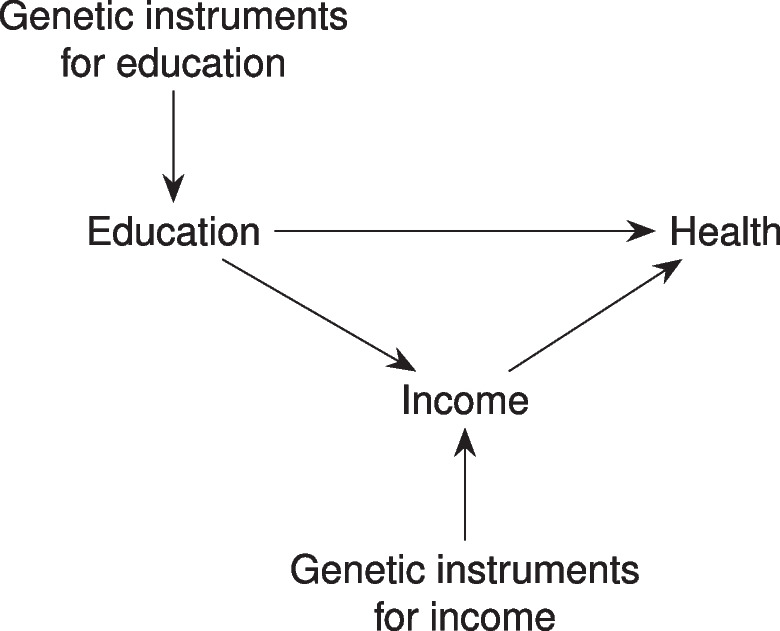
Causal graph showing the direction of the relationship between the education and income phenotypes assumed in the interpretation of the multivariable MR analysis

We used MVMR to investigate whether the effects of income could be explained by pleiotropic direct effects of education (Fig. 2). If the apparent effect of the income phenotype on health is primarily due to pleiotropic direct effects of education, we would expect the MVMR estimate for income to be attenuated compared to the single-variable MR analyses, while the education estimate would not be. Conversely, if the effect of the education phenotype is primarily mediated by income, we would expect the MVMR estimate for education to be attenuated.

We conducted MVMR using the *MVMR* package [64]. We included all SNPs that were significant at $p<5\times {10}^{-8}$ in either the income or education GWAS. We clumped the resulting set of SNPs using PLINK 1.90 [62], the 1000 Genomes reference population [63], an ${R}^{2}$ threshold of 0.001, and a window of 10,000 kb. In each clump, the SNP with the lowest p-value in the income GWAS was kept. To judge instrument strength, the conditional *F*-statistic for each exposure was calculated using the ‘strength_mvmr’ function.

### Steiger filtering and bidirectional analyses

Another potential source of bias in the primary analyses is misspecification of the primary phenotype [38]. If a health outcome affects income, then it is possible that the income GWAS has detected variants which affect income via the health outcome, rather than first income and subsequently the health outcome. We investigated the possibility of misspecification using two approaches: bidirectional MR and Steiger filtering (the latter only for continuous outcomes). Steiger filtering involves comparing how much of the variance in the exposure and the outcome is explained by each SNP, and excluding any SNPs that account for more variance in the outcome than the exposure. If the exposure GWAS identified SNPs whose effect on income was mediated by the outcome, we would expect Steiger filtering to substantially reduce the number of SNPs and attenuate the apparent effect estimates. We ran IVW analyses first using all SNPs as instruments, and then using the filtered set. We then repeated this procedure in the opposite direction, using genome-wide significant SNPs from the outcome GWAS as instruments for each health outcome, and estimating its effect on the income phenotype.

To perform Steiger filtering, we estimated the ${R}^{2}$ for each SNP in the exposure and outcome datasets using the ‘get_r_from_bsen’ function from the *TwoSampleMR* package [65]. We then removed all SNPs where ${R}^{2}$ for the outcome was greater than for the exposure. MR analyses were then conducted as per the main analysis. These analyses were all repeated in the opposite direction, i.e., to estimate the effect of the outcome on the exposure. Steiger filtering was only performed for continuous outcomes.

### Within-family Mendelian randomisation

Finally, we repeated the primary analyses using sibship-adjusted summary data for individual log income and five outcomes (depression, subjective wellbeing, BMI, alcohol consumption, and ever-smoking) from a GWAS that meta-analysed data from up to 18 European-ancestry cohorts of siblings (Additional file 2: Table S5) [66]. The purpose of this analysis was to account for population stratification, assortative mating, and dynastic effects [32]. Since pairs of siblings share genetic parents, and typically household environment, controlling for these factors should in principle reduce the influence of parental genotypes.

We considered as potential instruments all SNPs that were identified as significant ($p<5\times {10}^{-8}$) in the GWAS by Kweon and colleagues [22], as well as present in the sibship-adjusted exposure and outcome datasets. We then clumped these using PLINK and conducted MR analyses as in the main analysis.

## Results

### Primary Mendelian randomisation analyses

**Fig. 3:**
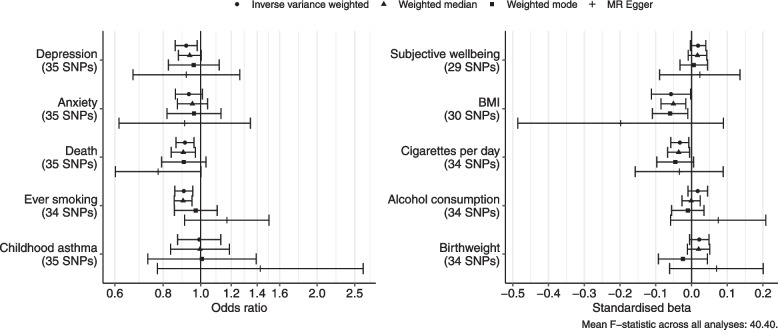
Forest plot showing effect estimates from primary Mendelian randomisation analyses (effect of a 10% increase in income)

The results from the primary MR analyses are shown in Fig. 3. In the IVW analysis, a 10% increase in income reduced the odds of depression diagnosis by 8.3% (95% confidence interval (CI) 2.1% to 14%), odds of anxiety disorder by 6.8% (95% CI 14% decrease to 1% increase), odds of death by 8.9% (95% CI 3.9% to 13.7%), and odds of ever smoking by 9.5% (95% CI 4.5% to 14.3%). It also reduced BMI by 0.06 SD (95% CI 0.003 to 0.11), and cigarettes consumed per day by 0.03 SD (95% CI 0.008 to 0.06). The effect on subjective well-being was small but consistently positive across estimators, with IVW estimating a 0.02 SD increase (95% CI − 0.003 to 0.04) for a 10% increase in income. There was no clear evidence of an effect on alcohol consumption or the negative control outcomes of birth weight and childhood asthma. MR scatterplots and funnel plots for each outcome are reported in Additional file 2: Fig. S1–S10.

The MR-Egger intercept coefficient was negligible and non-significant, showing little evidence of directional pleiotropy, for all outcomes except ever-smoking (*p* = 0.041) (Additional file 2: Table S1). For the non-null results, the effect direction was consistent across the robust estimators. The CAUSE analysis suggested a causal effect of income on depression (*p* = 0.030), BMI (*p* = 0.028), ever-smoking (*p* < 0.001), and cigarettes per day (*p* = 0.015), but not for the other outcomes (*p* > 0.1) (Additional file 2: Table S3).

### Multivariable Mendelian randomisation including education

When the SNPs for education [40] and income [22] were combined, tests for instrument strength showed conditional *F*-statistics of 1.27 (income) and 1.31 (education), well below the conventional threshold of 10. Consequently, these analyses were inconclusive, but are reported in Additional file 2: Table S2. There was also considerable overlap between the SNPs for both phenotypes: out of 36 instruments significantly associated with income, 29 were also significantly associated with education.

### Steiger filtering and bidirectional analyses

In the Steiger-filtered analyses, there was little evidence that misspecification of the exposure explained the results for continuous outcomes, and effect estimates were largely unaffected by Steiger filtering. Bidirectional analyses suggested a stronger effect of income on most outcomes than the reverse, although some of these analyses lacked precision due to the limited number of instruments available for the outcome (Additional file 2: Table S4).

### Within-family Mendelian randomisation

**Fig. 4:**
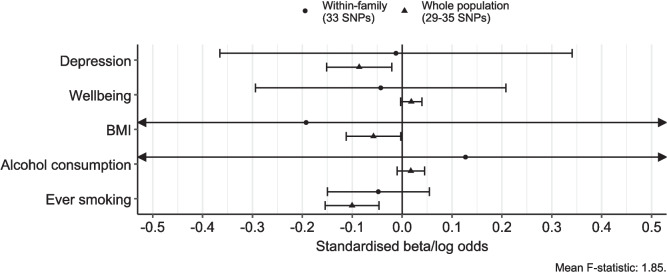
Forest plot showing effect estimates from within-family Mendelian randomisation analyses (effect of a 10% increase in income, in terms of standard deviations or log odds of the outcome)

As seen in Fig. 4, the within-family analyses were too imprecise to draw any meaningful conclusions. Sibship-adjusted data for negative control outcomes were not available.

## Discussion

We used two-sample Mendelian randomisation to estimate the causal effects of income on multiple health outcomes, using genetic variants associated with income, supplemented by sensitivity analyses including within-family and multivariable analyses to investigate potential biases. Our analyses suggested that higher income resulted in lower rates of depression, death, smoking, and lower BMI. Results also suggested a possible beneficial effect on anxiety, similar in magnitude to the effect on depression but less precisely estimated. Evidence was broadly consistent across multiple estimators, but the CAUSE method suggested that the effects on anxiety and death may be due to pleiotropy. We found little evidence of effects on alcohol consumption and subjective wellbeing. Although we attempted within-family analysis and multivariable analysis using income and education as exposures, these analyses were imprecise, placing considerable caveats on any causal conclusions.

Interpreting the size (not just the sign) of MR effect estimates requires a fourth instrumental variable assumption in addition to the core instrumental variable assumptions described in the introduction (the so-called point-identifying assumption [38]). One commonly used assumption is monotonicity, i.e., that the genetic variants associated with income will always increase, never decrease, income. This assumption would identify a weighted average of effects where each participant’s contribution was weighted by how influenced they were by the income score [67]. Although income was measured at a single time point in each individual (UK Biobank participants were enrolled at 40–69 years old [22]), MR estimates reflect the effect of differences in exposure or liability to exposure across the entire life course [27].

Since individual income is typically highly correlated with parental income (particularly in the UK, where the exposure GWAS was conducted [68]), individuals with higher genetic liability to income who are raised by their genetic parents will likely be exposed to higher household income prior to becoming earners themselves. Hence, the effects seen in our study likely reflect both the ‘indirect’ pathway via household income in childhood, and the ‘direct’ pathway via own income in adulthood. In principle, family-adjusted analyses with sufficient power may make it possible to estimate the effect through each of these two pathways separately, but as we have seen currently available family-based datasets are not sufficiently large.

We included two outcomes (birth weight and childhood asthma) which can by definition only be affected by exposures in childhood rather than adulthood. An effect on these outcomes would be attributable to either the indirect pathway or other biases (such as pleiotropy), but we found no evidence of an effect. This does not exclude the possibility of childhood or parental circumstances contributing substantially to the other results, however. Whether birth weight and asthma are suitable for use as negative controls [46, 47] also depends on whether parental socioeconomic circumstances indeed have a causal effect on these outcomes in the population studied, which is possible but not certain [69, 70]

Our findings are broadly consistent with prior literature on the causal effects of income on health. We found the strongest evidence of genuine causal effects on depression, BMI, and smoking. This is in line with existing evidence, which has more consistently supported the existence of a causal effect on mental health outcomes [69, 71, 72] than on physical health outcomes such as mortality [73, 74]. Smoking and obesity both have a well-known cross-sectional association with low socioeconomic position, but it has been disputed whether this is best explained by causal effects of income or by health selection mechanisms [75, 76]; notably, our findings lend additional support to the hypothesis that income causally affects health. We saw no effect of income on alcohol consumption, and indeed this is consistent with the lack of a cross-sectional association [77].

In comparing our results to the wider literature, it is important to note that studies often differ substantially in how they define income, and what causal contrast their estimates refer to [28]: for example, whether they use individual or household income [78], earned or unearned income, whether they estimate the effect of one-off changes or sustained differences in level [79], and so on. Here we attempt to estimate the effect of differences in lifetime income level, an exposure that is rarely studied but may be more important to health than short-term changes [79].

We now note some important limitations. The phenotype measured in the exposure GWAS was individual log occupational income, imputed from occupational codes. This measure is likely to have more measurement error and less precision than incomes taken from administrative data, for example; however, MR is less affected by measurement error in the exposure than conventional observational methods [29]. Individual income may also be an inaccurate representation of financial resources relevant to health outcomes since it does not account for the income of partners or other household members; this issue may be particularly significant for women [78]. We focused on individual income primarily because the household income measure in the UK Biobank (used in previous GWASs) uses a five-point categorical scale, resulting in low precision and less interpretable effect estimates. However, future studies should investigate how different income definitions affect the results.

The practical relevance of effect estimates from MR fundamentally depends on whether the mechanisms through which the genetic variants operate are comparable to real-world interventions on the exposure, a concept known as ‘gene-environment equivalence’ [38], and it is not clear whether this is the case for income. We also observed a large overlap between the education and income phenotypes, with many SNPs significantly associated with both. This overlap suggests a close relationship between the mechanisms involved for education and income, but more precise GWAS estimates would be required to investigate this relationship more closely. The plausibility of genetic instruments for education has been strengthened by triangulation studies comparing these with other natural experiment approaches [17]. Similar studies focusing on income would be a valuable further test of the viability of this approach.

Sample overlap between the datasets used to estimate the exposure and outcome associations can cause bias in MR analyses [51]. We were able to avoid sample overlap for most outcomes. The only exceptions were subjective wellbeing (which included 14% individuals from the UK Biobank) and birth weight (47%). However, any bias from sample overlap would be upward, towards the unadjusted exposure–outcome association in the population. Since we only observed small or null effects on these outcomes, such bias is unlikely to explain our results.

It was not possible to investigate non-linear effects of income or differences between population subgroups (including genders). This is an important limitation, since the health effects of additional income may differ substantially at both the higher and lower ends of the income distribution. Analysis using individual-level data would be required to address this. Although we hypothesise that the exposure in this study can be interpreted as lifetime income level, it is also unclear which aspects of this exposure are most important; for example, during which parts of the life course income most strongly affects future health. Future studies using individual-level data with income measured at multiple time points would help investigate this further.

Regarding the generalisability of our results, we are also limited by the individual genetic cohorts used for the GWAS results we rely on, which were limited to European-ancestry individuals, and whose participants may not be representative of the general population. For example, UK Biobank participants tend to be older, have better health, and have higher socioeconomic position than the general population [80]. The findings may thus not be generalisable to low/middle-income countries, non-European populations, or more recent birth cohorts, for example. It is also important to note that our results cannot be interpreted as an average causal effect even within the specific population studied without additional assumptions [81].

Finally, the limited power in within-family analyses meant that we were not able to investigate how far the effects seen reflect the direct effect of one’s own earned income, versus the indirect effects of parental socioeconomic position. Similarly, we also cannot exclude the potential for bias from assortative mating and population stratification, which may be important sources of bias in samples of unrelated individuals [66, 82]. The low precision in the within-family analyses may be explained by inefficiencies intrinsic to the study design: sibship-adjusted GWASs can only make use of within-family variation in genotype, and hence they are limited not only to the subset of siblings within a sample but the subset of sibling pairs that have non-identical alleles for a given SNP.

## Conclusions

In conclusion, MR using samples of unrelated individuals suggested that higher incomes led to reductions in depression, smoking, BMI, and mortality. We used a range of sensitivity analyses to investigate the mechanisms that underlie these results, including robust MR estimators, within-family analysis, and MVMR. However, many of these sensitivity analyses were imprecise and consistent with the null and our primary analysis. Future research should use larger family-based genetic cohorts, or if possible more efficient methods to estimate the relative contribution of direct genetic effects of income versus other sources of bias such as assortative mating or dynastic effects. Conducting future studies in a single-sample context will open possibilities beyond what we have explored here: for example, investigating how the use of different income definitions may affect results [78] and triangulation with other observational methods within the same sample [17, 34]. It is also important that future studies articulate clearly what the purpose and limitations of these methods are, since, in isolation, genetic epidemiology may be interpreted within inaccurate or harmful narratives (such as the idea that social inequalities are innate or biologically determined). However, used in conjunction with other methods in a triangulation framework, MR may provide new insight into the long-term health effects of income, and hence the mechanisms by which health inequalities may arise.

## Supplementary Information

Additional file 1. STROBE-MR checklist.

Additional file 2. Additional tables and figures.

## Data Availability

The data and code required to reproduce the results of this study are openly available in DeSci at https://beta.dpid.org/303 and GitHub at https://github.com/igelstorm/income-two-sample-mr-analysis.
