# Supplementary material for: Investigating causal effects of income on health using two-sample Mendelian randomisation
Source: BMC Glob Public Health. 2025 Feb 10;3:12. doi: 10.1186/s44263-025-00130-4 (PMC11809080; doi:10.1186/s44263-025-00130-4)
Supplement: Supplementary file 2 — Additional file 2. Additional tables and figures. [file 44263_2025_130_MOESM2_ESM.pdf]

Investigating causal effects of income on health using two-sample Mendelian randomisation

Additional file 2

Erik Igelström[1,*] Marcus R. Munafò[2,3] Ben M. Brumpton[3,4] Neil M. Davies[2,3,4,5]
George Davey Smith[2,3] Pekka Martikainen[6,7,8] Desmond Campbell[1] Peter Craig[1]
Jim Lewsey[9] S. Vittal Katikireddi[1]

[1] MRC/CSO Social and Public Health Sciences Unit, School of Health and Wellbeing, University of Glasgow, Glasgow, UK
[2] MRC Integrative Epidemiology Unit (IEU), Bristol Medical School, University of Bristol, Bristol, UK
[3] Population Health Sciences, Bristol Medical School, University of Bristol, Bristol, UK
[4] K. G. Jebsen Center for Genetic Epidemiology, Department of Public Health and Nursing, Norwegian University of Science and Technology, Trondheim, Norway
[5] Department of Statistical Sciences, University College London, London, UK
[6] Population Research Unit, Faculty of Social Sciences, University of Helsinki, Helsinki, Finland
[7] The Max Planck Institute for Demographic Research, Rostock, Germany
[8] Max Planck – University of Helsinki Center for Social Inequalities in Population Health, Helsinki, Finland
[9] Health Economics and Health Technology Assessment, School of Health and Wellbeing, University of Glasgow, Glasgow, UK

[*] Correspondence: Erik Igelström <Erik.Igelstrom@glasgow.ac.uk>

List of Tables

S1 Results from Mendelian randomisation analyses of the effect of 10% higher income. 3
S2 Multivariable Mendelian randomisation estimates of the direct effects of income and education. 5
S3 Results of CAUSE test comparing ‘sharing’ and ‘causal’ models. 7
S4 Results from bidirectional Mendelian randomisation analyses using the inverse-variance weighted estimator, before and after using Steiger filtering to remove SNPs more strongly associated with the outcome than the exposure. 8
S5 Information about the GWAS estimates used for within-family analyses. 9
S6 Results from Mendelian randomisation analyses of the effect of 10% higher income, using within-family GWAS estimates. 10

List of Figures

S1 Scatterplot of the SNP-exposure (income) and SNP-outcome (Depression) associations (A) and funnel plot for the Mendelian randomisation estimates (B). 11
S2 Scatterplot of the SNP-exposure (income) and SNP-outcome (Anxiety) associations (A) and funnel plot for the Mendelian randomisation estimates (B). 12
S3 Scatterplot of the SNP-exposure (income) and SNP-outcome (Subjective wellbeing) associations (A) and funnel plot for the Mendelian randomisation estimates (B). 13
S4 Scatterplot of the SNP-exposure (income) and SNP-outcome (Death) associations (A) and funnel plot for the Mendelian randomisation estimates (B). 14
S5 Scatterplot of the SNP-exposure (income) and SNP-outcome (BMI) associations (A) and funnel plot for the Mendelian randomisation estimates (B). 15
S6 Scatterplot of the SNP-exposure (income) and SNP-outcome (Childhood asthma) associations (A) and funnel plot for the Mendelian randomisation estimates (B). 16
S7 Scatterplot of the SNP-exposure (income) and SNP-outcome (Birthweight) associations (A) and funnel plot for the Mendelian randomisation estimates (B). 17
S8 Scatterplot of the SNP-exposure (income) and SNP-outcome (Ever smoking) associations (A) and funnel plot for the Mendelian randomisation estimates (B). 18
S9 Scatterplot of the SNP-exposure (income) and SNP-outcome (Cigarettes per day) associations (A) and funnel plot for the Mendelian randomisation estimates (B). 19
S10 Scatterplot of the SNP-exposure (income) and SNP-outcome (Alcohol consumption) associations (A) and funnel plot for the Mendelian randomisation estimates (B). 20

Table S1: Results from Mendelian randomisation analyses of the effect of 10% higher income.

Outcome	Method	Estimate	95% CI	P	SNPs	I^2	Mean F
Depression	IVW	-0.086	-0.151, -0.021	0.010	35		40.07
	Weighted median	-0.065	-0.133, 0.003	0.062	35		40.07
	Weighted mode	-0.041	-0.192, 0.110	0.591	35		40.07
	MR-Egger	-0.085	-0.402, 0.232	0.600	35	88.3%	40.07
	MR-Egger (intercept)	0.000	-0.006, 0.006	0.994	35	88.3%	40.07
Anxiety	IVW	-0.070	-0.151, 0.010	0.086	35		40.07
	Weighted median	-0.049	-0.139, 0.041	0.285	35		40.07
	Weighted mode	-0.040	-0.201, 0.121	0.629	35		40.07
	MR-Egger	-0.095	-0.486, 0.296	0.633	35	88.2%	40.07
	MR-Egger (intercept)	0.000	-0.007, 0.008	0.899	35	88.2%	40.07
Subjective wellbeing	IVW	0.018	-0.003, 0.040	0.094	29		40.87
	Weighted median	0.017	-0.009, 0.043	0.207	29		40.87
	Weighted mode	0.006	-0.032, 0.045	0.750	29		40.87
	MR-Egger	0.023	-0.089, 0.136	0.686	29	45.7%	40.87
	MR-Egger (intercept)	0.000	-0.002, 0.002	0.931	29	45.7%	40.87
Death	IVW	-0.093	-0.147, -0.040	<0.001	35		40.07
	Weighted median	-0.103	-0.175, -0.031	0.005	35		40.07
	Weighted mode	-0.100	-0.233, 0.032	0.137	35		40.07
	MR-Egger	-0.253	-0.508, 0.002	0.052	35	88.3%	40.07
	MR-Egger (intercept)	0.003	-0.002, 0.008	0.210	35	88.3%	40.07
BMI	IVW	-0.057	-0.112, -0.003	0.039	30		41.25
	Weighted median	-0.051	-0.085, -0.016	0.004	30		41.25
	Weighted mode	-0.060	-0.109, -0.010	0.018	30		41.25
	MR-Egger	-0.198	-0.486, 0.089	0.176	30	96.7%	41.25
	MR-Egger (intercept)	0.003	-0.003, 0.008	0.328	30	96.7%	41.25
Childhood asthma	IVW	-0.008	-0.137, 0.120	0.898	35		40.07
	Weighted median	-0.004	-0.179, 0.171	0.964	35		40.07
	Weighted mode	0.008	-0.315, 0.330	0.963	35		40.07
	MR-Egger	0.355	-0.258, 0.967	0.256	35	88.2%	40.07
	MR-Egger (intercept)	-0.007	-0.019, 0.005	0.235	35	88.2%	40.07
Birthweight	IVW	0.022	-0.005, 0.049	0.117	34		40.39
	Weighted median	0.020	-0.011, 0.051	0.215	34		40.39
	Weighted mode	-0.024	-0.092, 0.044	0.491	34		40.39
	MR-Egger	0.070	-0.061, 0.201	0.297	34	24.0%	40.39
	MR-Egger (intercept)	-0.001	-0.003, 0.002	0.463	34	24.0%	40.39
Ever smoking	IVW	-0.100	-0.154, -0.046	<0.001	34		40.39
	Weighted median	-0.105	-0.158, -0.051	<0.001	34		40.39
	Weighted mode	-0.029	-0.156, 0.098	0.654	34		40.39
	MR-Egger	0.156	-0.095, 0.406	0.224	34	30.7%	40.39
	MR-Egger (intercept)	-0.005	-0.010, -0.000	0.041	34	30.7%	40.39
Cigarettes per day	IVW	-0.033	-0.058, -0.008	0.010	34		40.39
	Weighted median	-0.036	-0.067, -0.005	0.022	34		40.39
	Weighted mode	-0.046	-0.097, 0.006	0.082	34		40.39
	MR-Egger	-0.034	-0.158, 0.089	0.584	34	31.5%	40.39
	MR-Egger (intercept)	0.000	-0.002, 0.002	0.978	34	31.5%	40.39

(continued)

Outcome	Method	Estimate	95% CI	P	SNPs	I^2	Mean F
Alcohol consumption	IVW	0.017	-0.010, 0.045	0.212	34		40.39
	Weighted median	-0.001	-0.026, 0.025	0.942	34		40.39
	Weighted mode	-0.010	-0.056, 0.035	0.655	34		40.39
	MR-Egger	0.075	-0.058, 0.208	0.271	34	26.3%	40.39
	MR-Egger (intercept)	-0.001	-0.004, 0.001	0.388	34	26.3%	40.39

Note:

BMI: body mass index; CI: confidence interval; IVW: inverse-variance weighted; MR: Mendelian randomisation; SNP: single nucleotide polymorphism. Estimates have been rescaled to reflect the effect of a 10% increase in income. Estimates are expressed as log odds for binary outcomes (depression, anxiety, death, childhood asthma, ever smoking) and in standard deviation units for continuous outcomes (subjective wellbeing, BMI, birthweight, cigarettes per day, alcohol consumption).

Table S2: Multivariable Mendelian randomisation estimates of the direct effects of income and education.

Outcome	Method	Term	Estimate	95% CI	P	SNPs	F statistic
Depression	IVW	Income	-0.189	-0.561, 0.184	0.321	320	1.26
		Education	-0.130	-0.480, 0.220	0.465	320	1.30
	Weighted median	Income	-0.228	-0.587, 0.132	0.214	320	1.26
		Education	-0.018	-0.362, 0.326	0.919	320	1.30
	MR-Egger	Income	-0.205	-0.581, 0.171	0.286	320	1.26
		Education	-0.254	-0.757, 0.250	0.323	320	1.30
		(Intercept)	0.002	-0.003, 0.007	0.504	320	
Anxiety	IVW	Income	-0.120	-0.576, 0.336	0.605	320	1.26
		Education	-0.341	-0.769, 0.086	0.118	320	1.30
	Weighted median	Income	-0.239	-0.691, 0.213	0.301	320	1.26
		Education	-0.172	-0.612, 0.268	0.444	320	1.30
	MR-Egger	Income	-0.117	-0.577, 0.343	0.618	320	1.26
		Education	-0.316	-0.932, 0.299	0.314	320	1.30
		(Intercept)	0.000	-0.007, 0.006	0.911	320	
Subjective wellbeing	IVW	Income	0.036	-0.088, 0.160	0.568	300	1.24
		Education	0.046	-0.069, 0.162	0.433	300	1.28
	Weighted median	Income	0.024	-0.110, 0.158	0.725	300	1.24
		Education	0.058	-0.068, 0.184	0.369	300	1.28
	MR-Egger	Income	0.028	-0.096, 0.152	0.661	300	1.24
		Education	-0.036	-0.209, 0.138	0.686	300	1.28
		(Intercept)	0.001	-0.001, 0.003	0.215	300	
Death	IVW	Income	-0.018	-0.335, 0.299	0.913	320	1.26
		Education	-0.313	-0.611, -0.016	0.039	320	1.30
	Weighted median	Income	-0.166	-0.528, 0.196	0.368	320	1.26
		Education	-0.223	-0.570, 0.125	0.209	320	1.30
	MR-Egger	Income	-0.024	-0.344, 0.296	0.885	320	1.26
		Education	-0.359	-0.787, 0.069	0.100	320	1.30
		(Intercept)	0.001	-0.004, 0.005	0.770	320	
BMI	IVW	Income	-0.125	-0.327, 0.078	0.228	297	1.22
		Education	-0.103	-0.292, 0.085	0.283	297	1.24
	Weighted median	Income	-0.013	-0.192, 0.166	0.885	297	1.22
		Education	-0.216	-0.384, -0.048	0.012	297	1.24
	MR-Egger	Income	-0.110	-0.314, 0.094	0.290	297	1.22
		Education	0.002	-0.270, 0.273	0.991	297	1.24
		(Intercept)	-0.002	-0.004, 0.001	0.292	297	
Childhood asthma	IVW	Income	0.233	-0.556, 1.021	0.563	320	1.26
		Education	-0.641	-1.381, 0.099	0.090	320	1.30
	Weighted median	Income	-0.109	-0.970, 0.753	0.804	320	1.26
		Education	-0.172	-0.994, 0.649	0.681	320	1.30
	MR-Egger	Income	0.209	-0.587, 1.004	0.607	320	1.26
		Education	-0.828	-1.892, 0.237	0.127	320	1.30
		(Intercept)	0.003	-0.008, 0.014	0.632	320	

(continued)

Outcome	Method	Term	Estimate	95% CI	P	SNPs	F statistic
Birthweight	IVW	Income	-0.024	-0.181, 0.133	0.764	323	1.27
		Education	0.134	-0.014, 0.282	0.075	323	1.31
	Weighted median	Income	-0.090	-0.251, 0.072	0.276	323	1.27
		Education	0.189	0.036, 0.341	0.015	323	1.31
	MR-Egger	Income	-0.010	-0.169, 0.148	0.898	323	1.27
		Education	0.236	0.025, 0.447	0.029	323	1.31
		(Intercept)	-0.001	-0.004, 0.001	0.187	323	
Ever smoking	IVW	Income	-0.110	-0.359, 0.139	0.385	323	1.27
		Education	-0.384	-0.617, -0.151	0.001	323	1.31
	Weighted median	Income	-0.072	-0.325, 0.181	0.576	323	1.27
		Education	-0.415	-0.654, -0.176	<0.001	323	1.31
	MR-Egger	Income	-0.103	-0.355, 0.148	0.421	323	1.27
		Education	-0.334	-0.668, 0.001	0.051	323	1.31
		(Intercept)	-0.001	-0.004, 0.003	0.679	323	
Cigarettes per day	IVW	Income	-0.100	-0.250, 0.050	0.190	323	1.27
		Education	-0.066	-0.207, 0.074	0.355	323	1.31
	Weighted median	Income	-0.089	-0.247, 0.069	0.269	323	1.27
		Education	-0.068	-0.216, 0.081	0.370	323	1.31
	MR-Egger	Income	-0.091	-0.242, 0.060	0.239	323	1.27
		Education	0.000	-0.202, 0.201	0.997	323	1.31
		(Intercept)	-0.001	-0.003, 0.001	0.369	323	
Alcohol consumption	IVW	Income	0.046	-0.072, 0.163	0.448	323	1.27
		Education	-0.011	-0.122, 0.099	0.841	323	1.31
	Weighted median	Income	-0.045	-0.168, 0.077	0.469	323	1.27
		Education	0.057	-0.059, 0.173	0.331	323	1.31
	MR-Egger	Income	0.042	-0.077, 0.161	0.485	323	1.27
		Education	-0.035	-0.193, 0.123	0.664	323	1.31
		(Intercept)	0.000	-0.001, 0.002	0.681	323	

Note:
BMI: body mass index; CI: confidence interval; IVW: inverse-variance weighted; MR: Mendelian randomisation; SNP: single nucleotide polymorphism. Estimates reflect the effect of a 1 standard deviation increase in log income or years of education. Estimates are expressed as log odds for binary outcomes (depression, anxiety, death, childhood asthma, ever smoking) and in standard deviation units for continuous outcomes (subjective wellbeing, BMI, birthweight, cigarettes per day, alcohol consumption).

Table S3: Results of CAUSE test comparing ‘sharing’ and ‘causal’ models.

Outcome	ΔELPD	SE of ΔELPD	Z	p
Depression	-3.568	1.897	-1.880	0.030
Anxiety	-1.988	1.666	-1.193	0.116
Subjective wellbeing	0.455	0.596	0.764	0.777
Death	-1.972	1.692	-1.166	0.122
BMI	-3.530	1.841	-1.918	0.028
Childhood asthma	-1.409	1.334	-1.056	0.145
Birthweight	-1.086	1.363	-0.797	0.213
Ever smoking	-6.078	1.664	-3.653	<0.001
Cigarettes per day	-3.629	1.667	-2.177	0.015
Alcohol consumption	0.785	0.199	3.942	>0.999

Note:

BMI: body mass index; ELPD: expected log predictive density; SE: standard error; SNP: single nucleotide polymorphism.

Table S4: Results from bidirectional Mendelian randomisation analyses using the inverse-variance weighted estimator, before and after using Steiger filtering to remove SNPs more strongly associated with the outcome than the exposure.

		All SNPs			After Steiger filtering		
Exposure	Outcome	Estimate	95% CI	SNPs	Estimate	95% CI	SNPs
Income	Depression	-0.317	-0.557, -0.077	35			
Depression	Income	-0.096	-0.194, 0.001	8			
Income	Anxiety	-0.259	-0.555, 0.037	35			
Anxiety	Income	0.004	-0.100, 0.109	1			
Income	Subjective wellbeing	0.067	-0.011, 0.146	29	0.066	-0.031, 0.163	15
Subjective wellbeing	Income	-0.174	-0.465, 0.118	1			
Income	Death	-0.344	-0.541, -0.147	35			
Death	Income	-0.011	-0.073, 0.051	2			
Income	BMI	-0.212	-0.413, -0.010	30	-0.110	-0.344, 0.125	17
BMI	Income	-0.055	-0.106, -0.004	67	-0.034	-0.103, 0.034	39
Income	Ever smoking	-0.370	-0.568, -0.171	34			
Ever smoking	Income	-0.203	-0.352, -0.054	9			
Income	Cigarettes per day	-0.121	-0.213, -0.028	34	-0.131	-0.247, -0.016	19
Cigarettes per day	Income	-0.071	-0.136, -0.005	9	-0.002	-0.107, 0.102	4
Income	Alcohol consumption	0.064	-0.037, 0.165	34	0.070	-0.054, 0.195	20
Alcohol consumption	Income	-0.132	-0.380, 0.116	6	-0.132	-0.380, 0.116	6
Income	Birthweight	0.080	-0.020, 0.179	34	0.089	-0.053, 0.232	19
Birthweight	Income	0.057	0.026, 0.088	48	0.038	-0.010, 0.087	21
Income	Childhood asthma	-0.031	-0.504, 0.443	35			
Childhood asthma	Income	-0.016	-0.026, -0.006	10			

Note:
BMI: body mass index; CI: confidence interval; MR: Mendelian randomisation; SNP: single nucleotide polymorphism. Exposures and outcomes are measured in log odds for binary phenotypes (depression, anxiety, death, childhood asthma, ever smoking) and in standard deviation units for continuous phenotypes (log income, subjective wellbeing, BMI, birthweight, cigarettes per day, alcohol consumption). Steiger filtering was not performed for binary outcomes.

Table S5: Information about the GWAS estimates used for within-family analyses.

Phenotype	OpenGWAS ID	Sample size
Income		45,603
Depressive symptoms	ieu-b-4839	16,782
Subjective wellbeing	ieu-b-4851	22,656
Body mass index	ieu-b-4815	51,852
Alcohol consumption	ieu-b-4833	29,540
Ever smoking	ieu-b-4857	44,052

Table S6: Results from Mendelian randomisation analyses of the effect of 10% higher income, using within-family GWAS estimates.

Outcome	Method	Estimate	95% CI	P	SNPs	Mean F
Depressive symptoms	IVW	-0.012	-0.365, 0.341	0.945	33	1.85
Subjective wellbeing	IVW	-0.043	-0.294, 0.208	0.738	33	1.85
BMI	IVW	-0.192	-1.422, 1.037	0.759	33	1.85
Alcohol consumption	IVW	0.127	-0.648, 0.902	0.748	33	1.85
Ever smoking	IVW	-0.048	-0.150, 0.054	0.360	33	1.85

Note:
BMI: body mass index; CI: confidence interval; IVW: inverse-variance weighted; SNP: single nucleotide polymorphism. Estimates have been rescaled to reflect the effect of a 10% increase in income in terms of standard deviations of the outcome.

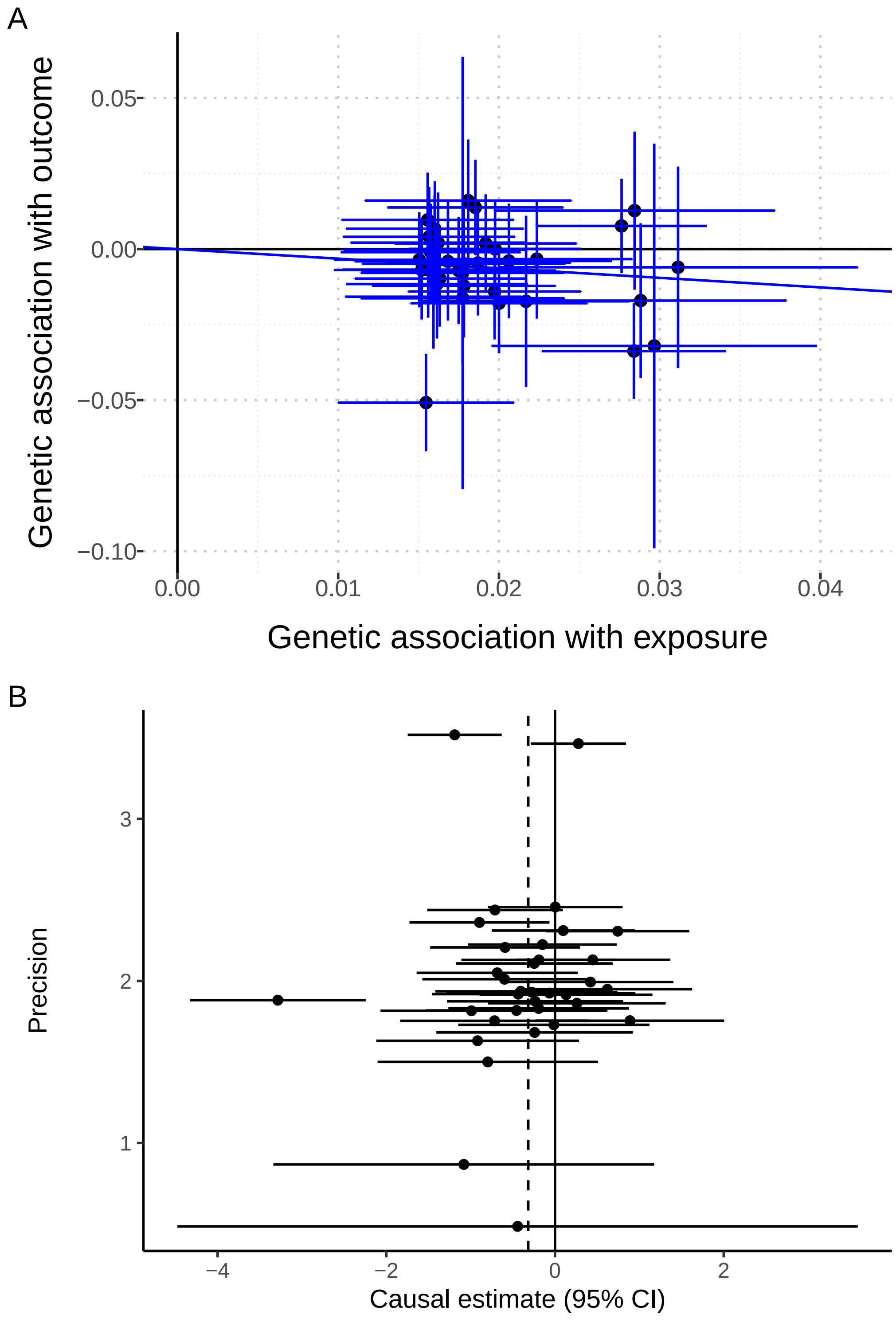

Figure S1: Scatterplot of the SNP-exposure (income) and SNP-outcome (Depression) associations (A) and funnel plot for the Mendelian randomisation estimates (B).

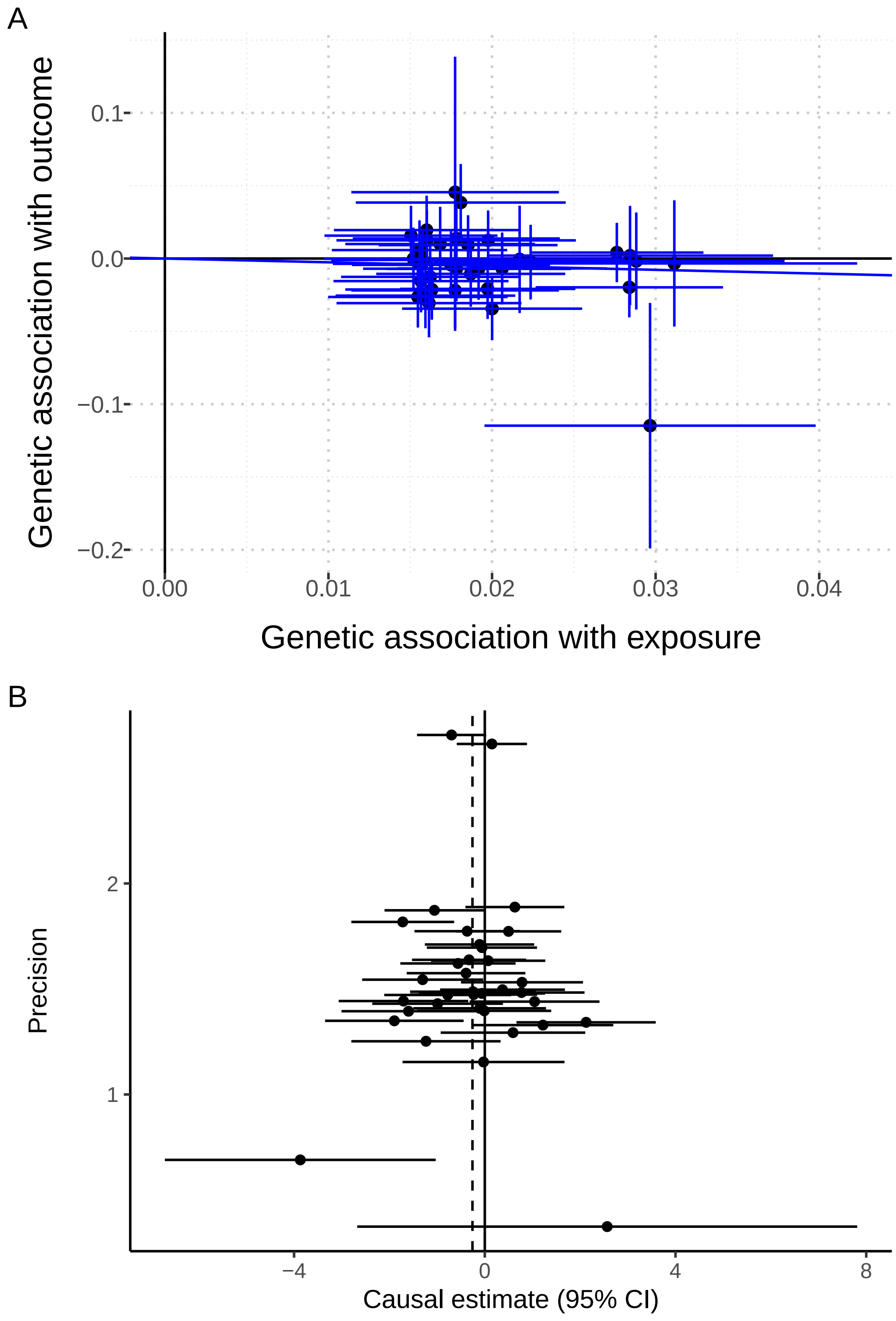

Figure S2: Scatterplot of the SNP-exposure (income) and SNP-outcome (Anxiety) associations (A) and funnel plot for the Mendelian randomisation estimates (B).

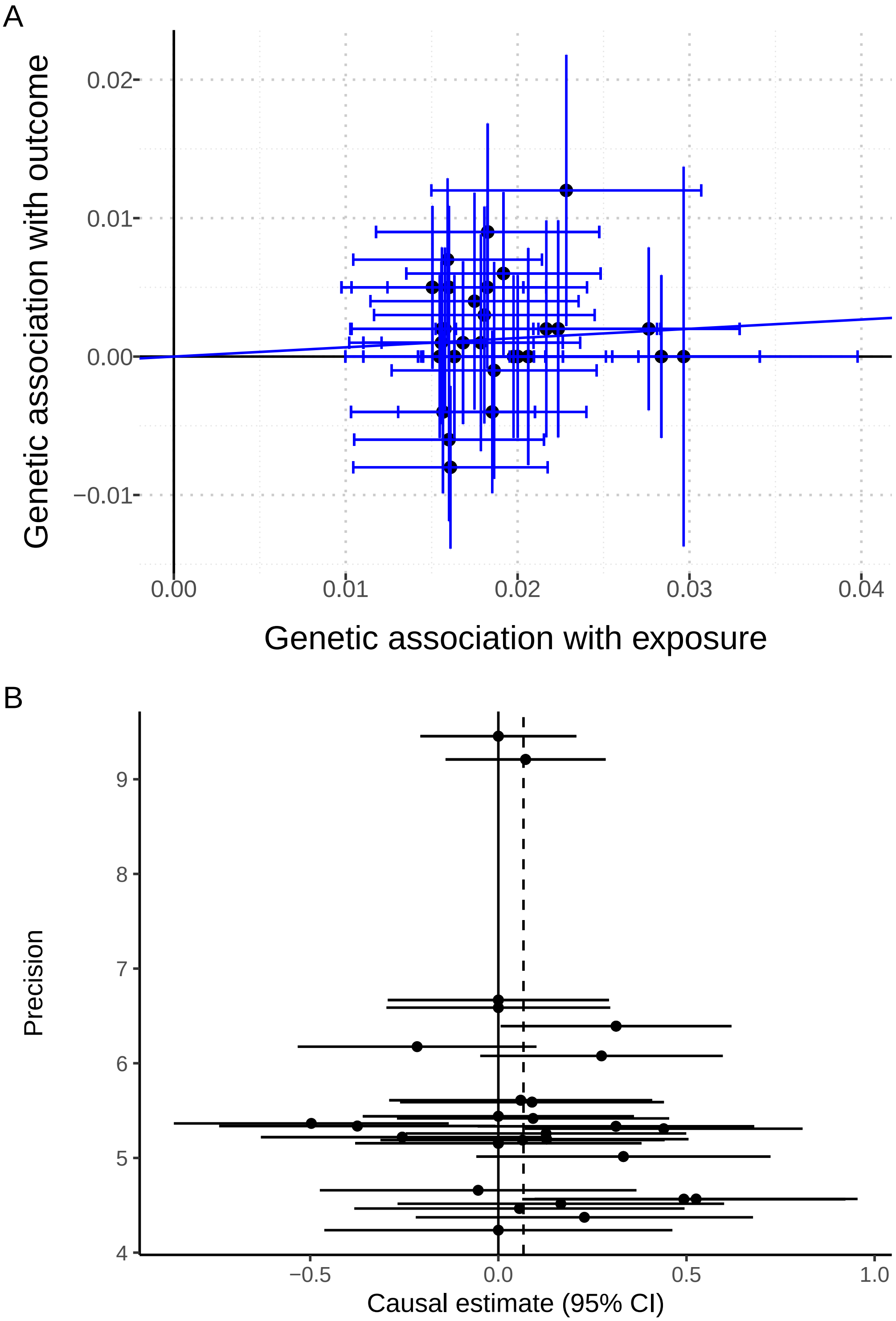

Figure S3: Scatterplot of the SNP-exposure (income) and SNP-outcome (Subjective wellbeing) associations (A) and funnel plot for the Mendelian randomisation estimates (B).

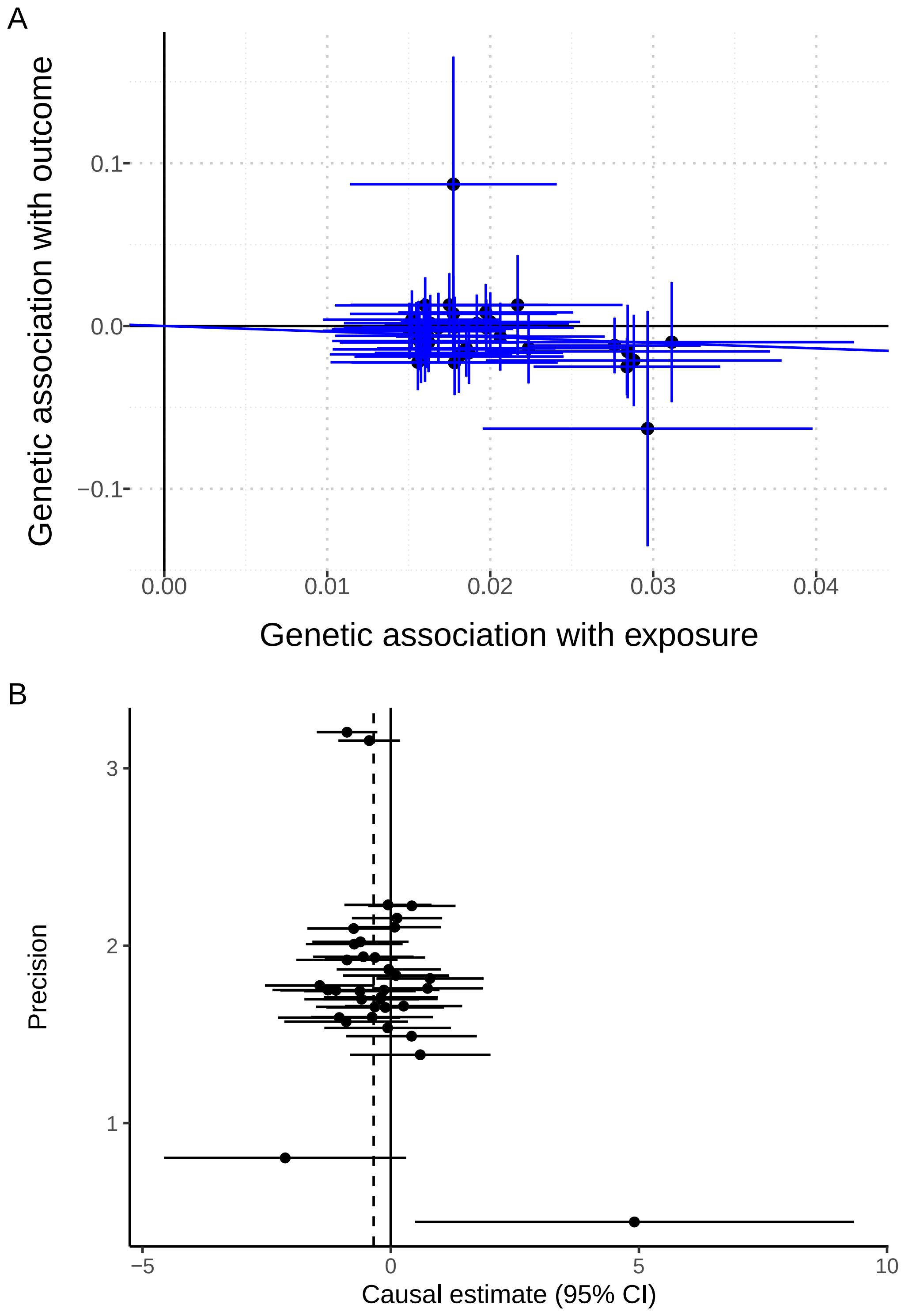

Figure S4: Scatterplot of the SNP-exposure (income) and SNP-outcome (Death) associations (A) and funnel plot for the Mendelian randomisation estimates (B).

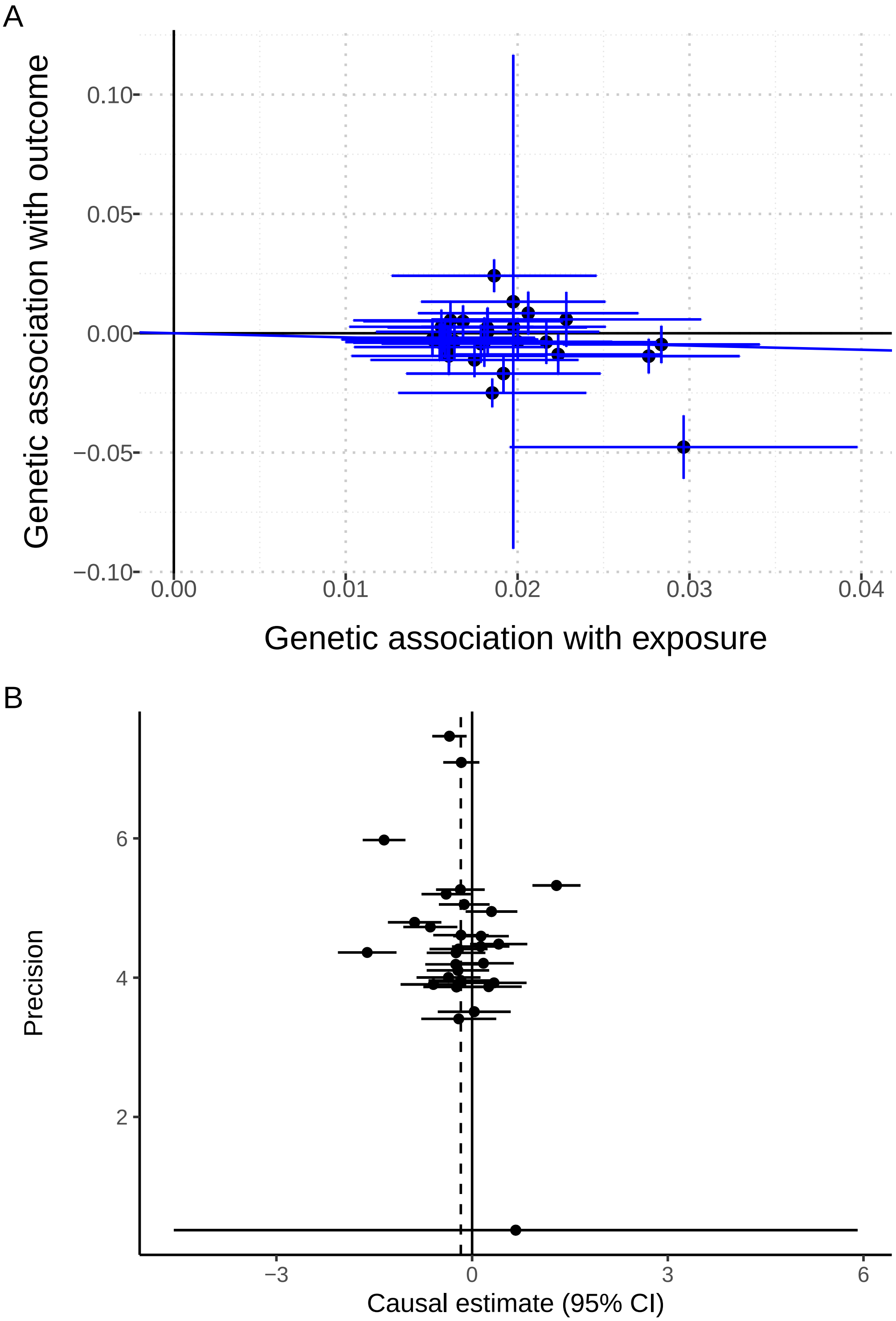

Figure S5: Scatterplot of the SNP-exposure (income) and SNP-outcome (BMI) associations (A) and funnel plot for the Mendelian randomisation estimates (B).

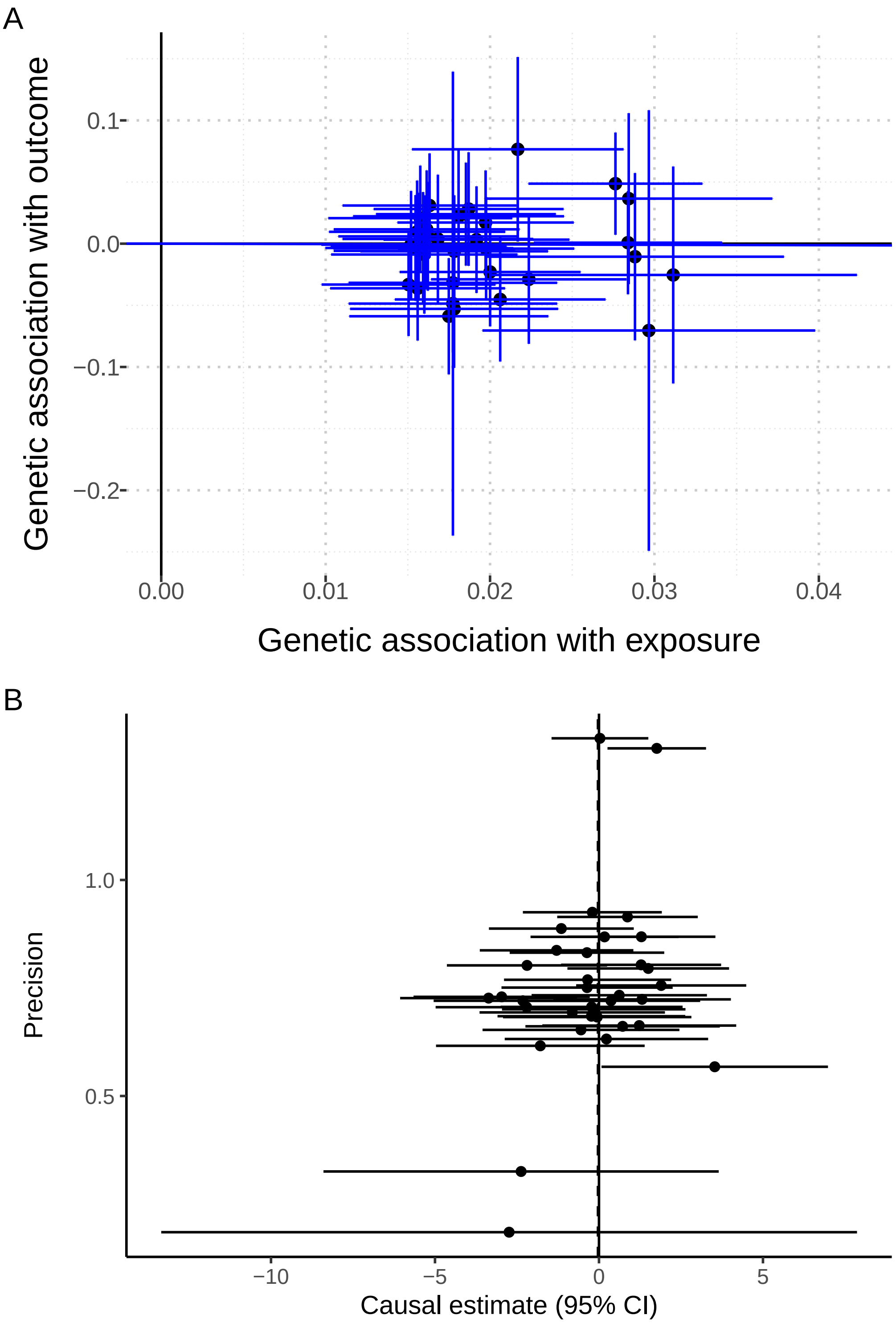

Figure S6: Scatterplot of the SNP-exposure (income) and SNP-outcome (Childhood asthma) associations (A) and funnel plot for the Mendelian randomisation estimates (B).

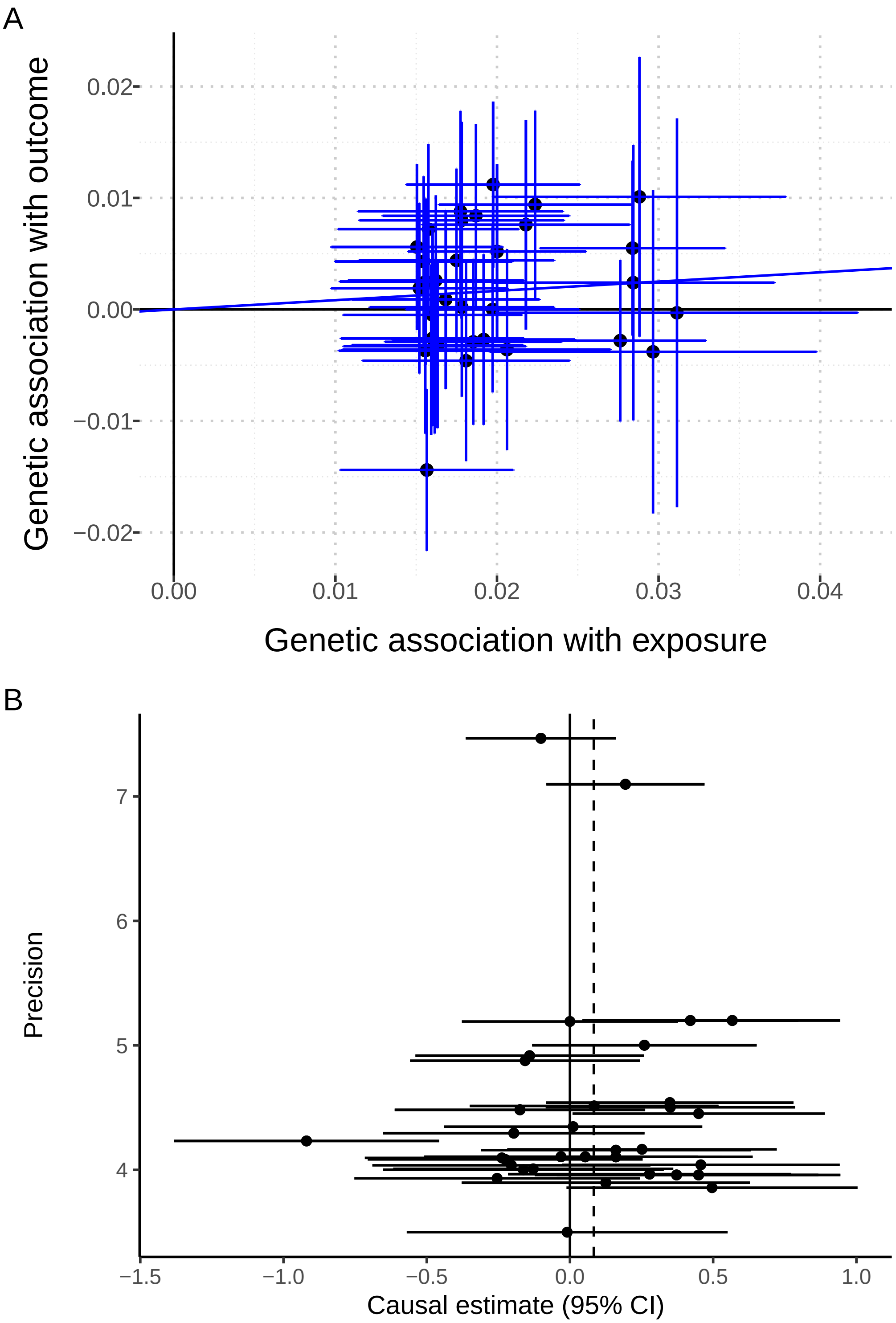

Figure S7: Scatterplot of the SNP-exposure (income) and SNP-outcome (Birthweight) associations (A) and funnel plot for the Mendelian randomisation estimates (B).

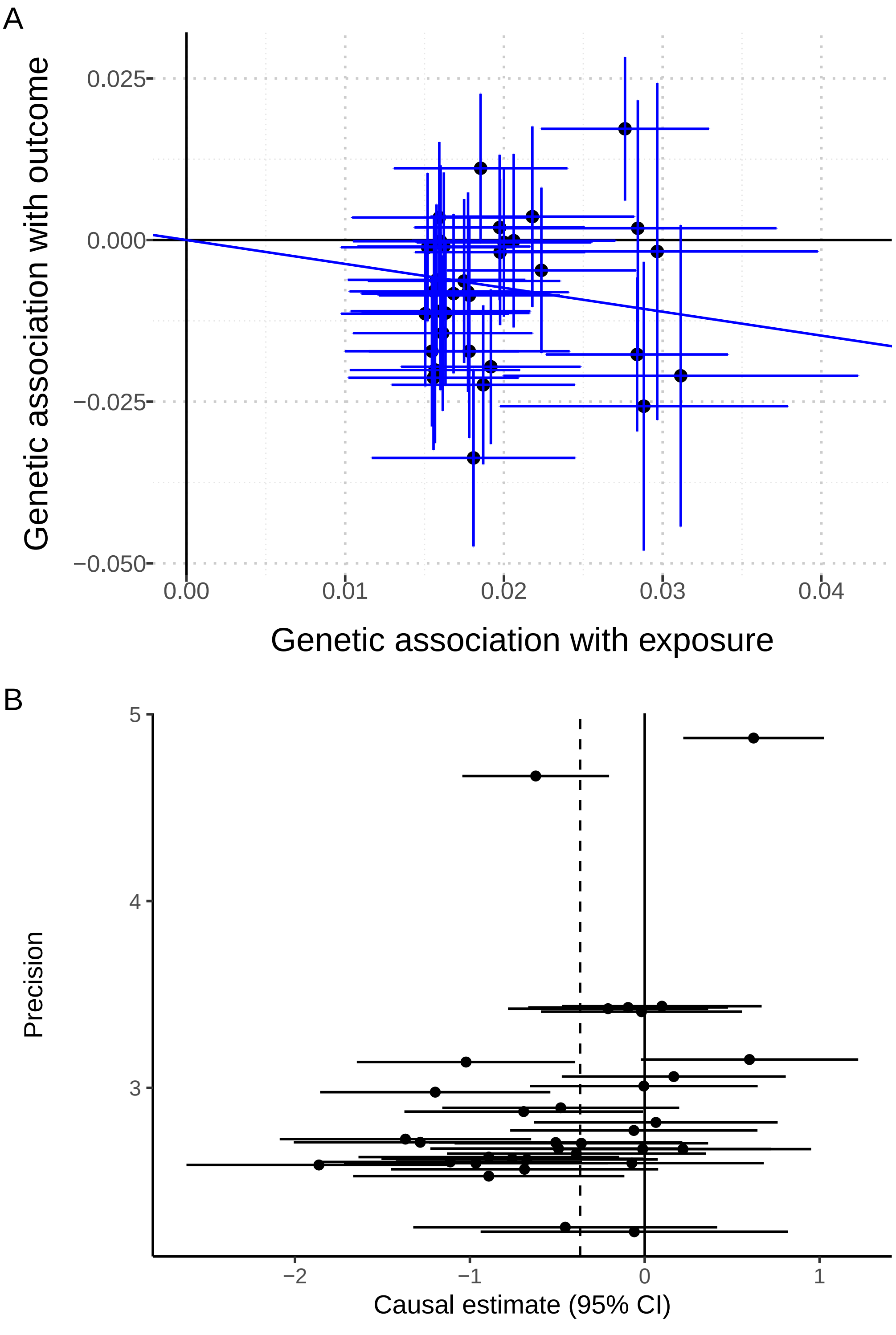

Figure S8: Scatterplot of the SNP-exposure (income) and SNP-outcome (Ever smoking) associations (A) and funnel plot for the Mendelian randomisation estimates (B).

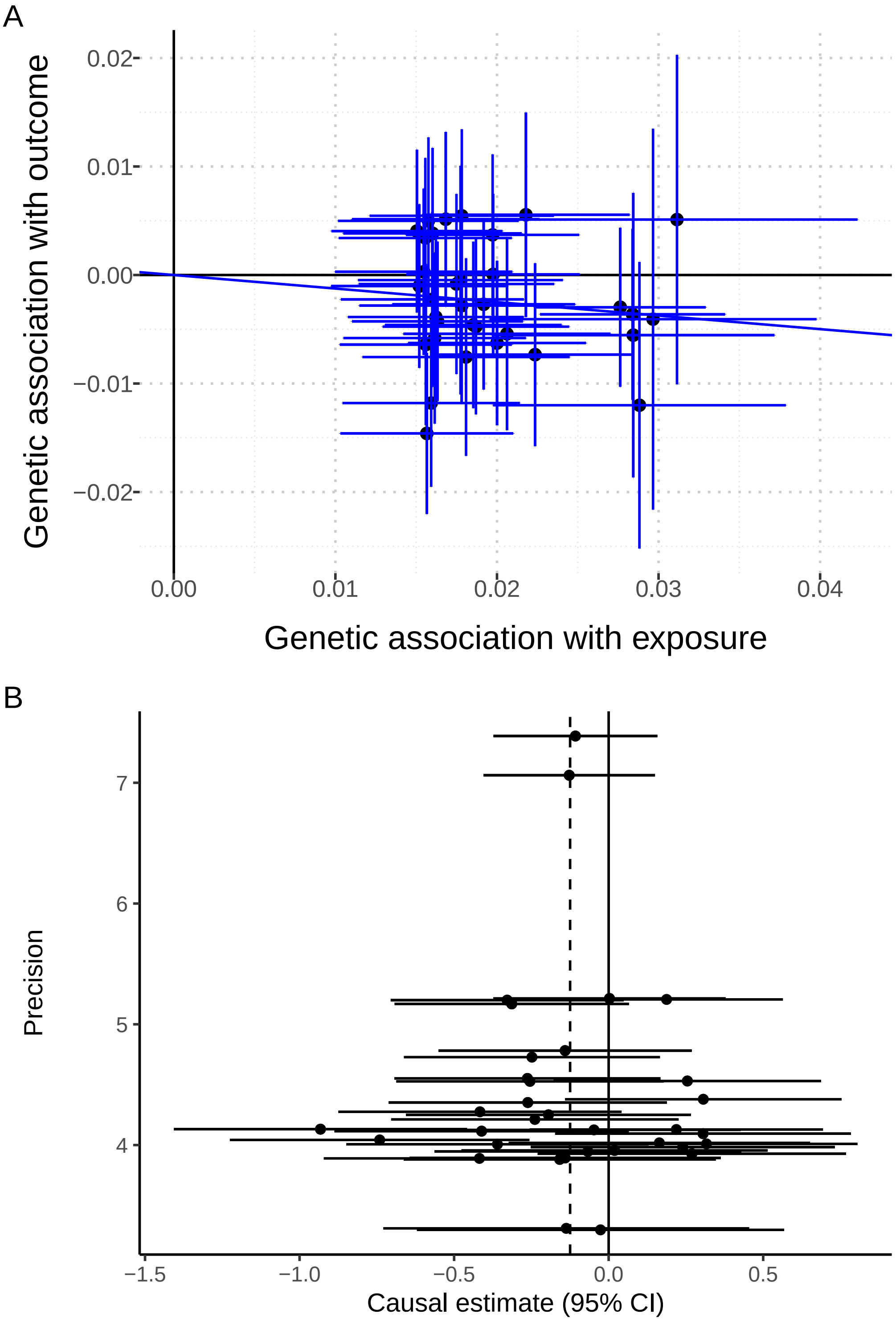

Figure S9: Scatterplot of the SNP-exposure (income) and SNP-outcome (Cigarettes per day) associations (A) and funnel plot for the Mendelian randomisation estimates (B).

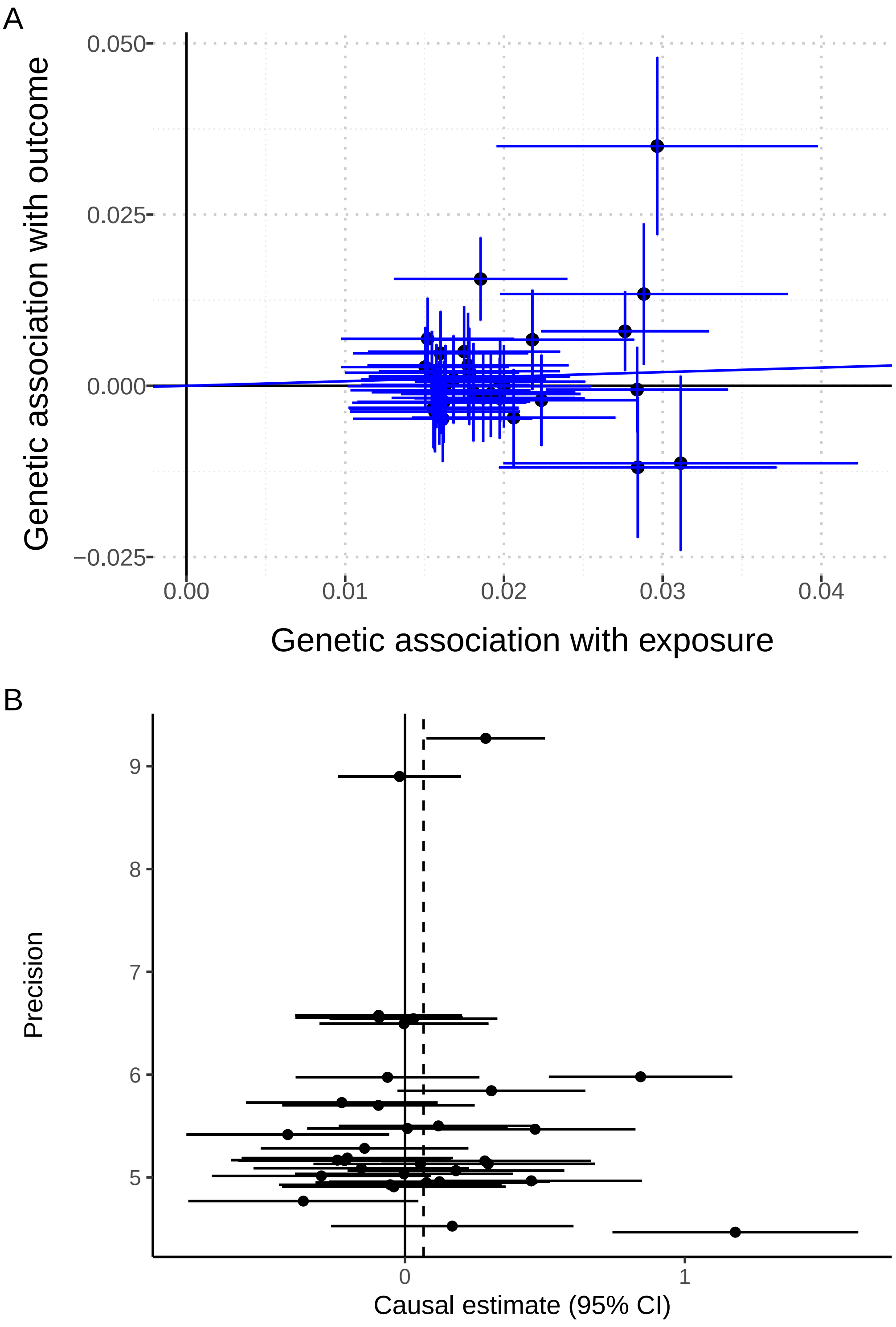

Figure S10: Scatterplot of the SNP-exposure (income) and SNP-outcome (Alcohol consumption) associations (A) and funnel plot for the Mendelian randomisation estimates (B).
